# Catalytic Asymmetric Hydrogenation of 3-Substituted Benzisoxazoles

**DOI:** 10.3390/molecules17096901

**Published:** 2012-06-06

**Authors:** Ryuhei Ikeda, Ryoichi Kuwano

**Affiliations:** Department of Chemistry, Graduate School of Sciences, and International Research Center for Molecular Systems (IRCMS), Kyushu University, 6-10-1 Hakozaki, Higashi-ku, Fukuoka 812-8581, Japan

**Keywords:** ruthenium, catalytic asymmetric synthesis, hydrogenation, benzisoxazole, amine

## Abstract

A variety of 3-substituted benzisoxazoles were reduced with hydrogen using the chiral ruthenium catalyst, {RuCl(*p*-cymene)[(*R*,*R*)-(*S*,*S*)-PhTRAP]}Cl. The ruthenium-catalyzed hydrogenation proceeded in high yield in the presence of an acylating agent, affording *α*-substituted *o*-hydroxybenzylamines with up to 57% *ee*. In the catalytic transformation, the N–O bond of the benzisoxazole substrate is reductively cleaved by the ruthenium complex under the hydrogenation conditions. The C–N double bond of the resulting imine is saturated stereoselectively through the PhTRAP–ruthenium catalysis. The hydrogenation produces chiral primary amines, which may work as catalytic poisons, however, the amino group of the hydrogenation product is rapidly acylated when the reaction is conducted in the presence of an appropriate acylating agent, such as Boc_2_O or Cbz-OSu.

## 1. Introduction

**Figure 1:**
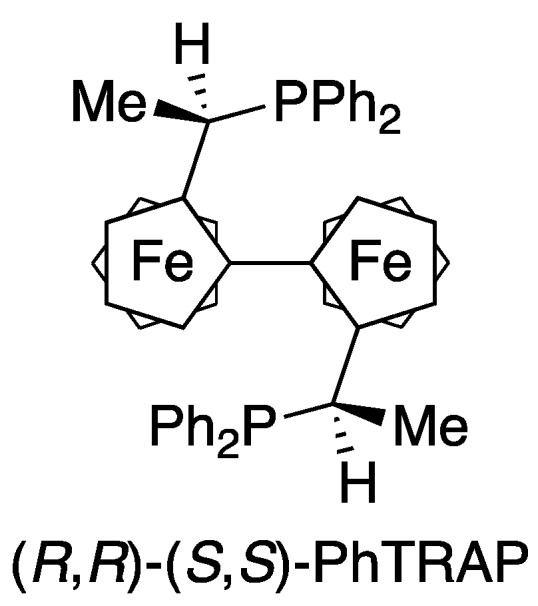
Structure of (*R*,*R*)-(*S*,*S*)-PhTRAP.

Catalytic asymmetric hydrogenation of heteroaromatics is an important issue in synthetic organic chemistry [1,2,3,4]. The reaction offers a straightforward access to optically active heterocycles, which are seen in various medicines as well as natural products. Thus, during the last decade the enantioselective reduction of heteroarenes has been studied intensively. High enantioselectivities have been achieved for the hydrogenation of quinolines [5,6,7,8,9], quinoxalines [10,11,12,13,14], pyridines [15,16,17], furans, and benzofurans [18,19]. Meanwhile, we have also directed our attention to the asymmetric hydrogenation of arenes, particularly nitrogen-containing 5-membered heteroaromatics [3]. In a series of our studies, a *trans*-chelating chiral bisphosphine, PhTRAP (Figure 1) [20,21], was mainly used as the chiral ligand. PhTRAP–rhodium or ruthenium complex allowed indoles [22,23,24,25,26,27] and pyrroles [28,29] to be reduced with hydrogen to the corresponding chiral indolines and pyrrolidines with high enantiomeric excesses, respectively. Moreover, the chiral ruthenium catalyst recently proved to be useful for the asymmetric hydrogenation of imidazoles and oxazoles, which contain two heteroatoms in their aromatic rings [30,31].

We conceived that PhTRAP–ruthenium complex might also catalyze the hydrogenation of 5-membered heteroarenes containing an N–O bond, such as isoxazoles and benzisoxazoles. If the hydrogenation of 3-substituted benzisoxazoles were to proceed with high enantioselectivity, it would provide optically active benzisoxazolines bearing a stereogenic center at the 3-position. The benzisoxazoline products can be transformed into optically active *α*-substituted *o*-hydroxy-benzylamines, because N–O bonds are known to break through heterogeneous catalysis under hydrogenation conditions [32,33]. Enantiomeric benzylamines are often used as chiral auxiliaries [34,35] and constituents of catalysts for asymmetric synthesis [36,37,38]. Furthermore, the structural motives are seen in many isoquinoline alkaloids [39,40,41,42]. Chiral amines are typically prepared through enzymatic [43] or chemical resolution of their racemates [44]. The diastereoselective nucleophilic additions to imines have been applied to the asymmetric synthesis of the chiral amines [45,46]. However, to our knowledge there have been only a few reports on the enantioselective synthesis of *α*-substituted *o*-hydroxybenzylamines [47,48]. In this paper, we report a catalytic asymmetric hydrogenation of 3-substituted benzisoxazoles to yield chiral *α*-substituted *o*-hydroxybenzylamines. The asymmetric reaction proceeded through the PhTRAP–ruthenium catalysis, which transformed the benzo-fused heteroaromatics into *α*-substituted *o*-hydroxybenzylamines in high yields and up to 57% *ee*.

## 2. Results and Discussion

### 2.1. Optimization of Reaction Conditions

In our initial attempts, 3-ethylbenzisoxazole (**1a**) was treated with pressurized hydrogen gas (50 atm) in toluene or isobutyl alcohol at 80 °C for 4 h in the presence of {RuCl(*p*-cymene)[(*R*,*R*)-(*S*,*S*)-PhTRAP]}Cl (**2**) (Table 1, entries 1 and 2) [25]. A small amount of **1a** reacted with hydrogen, but no saturation of the C–N double bond was observed in either reaction, which afforded only the achiral imine **3a**, formed through the hydrogenolytic cleavage of the N–O bond of **1a** [49,50]. The ruthenium catalyst failed to reduce the C–N double bond even in the presence of *N*,*N*,*N'*,*N'*-tetramethylguanidine (TMG) (entries 3 and 4), even though in our previous reports the base additive brought about a remarkable acceleration of hydrogenations of heteroaromatics [22,23,24,25,28,30,31]. We were pleased that the hydrogenation of the benzisoxazole was accompanied by the reduction of the C–N double bond in the presence of stoichiometric Boc_2_O, which afforded *N*-Boc-protected (*R*)-1-(2-hydroxyphenyl)-1-propylamine **4a** with 25% *ee* (entry 5). To our surprise, no formation of **4a** was observed in the reaction using both Boc_2_O and TMG (entry 6). The base additive might inhibit the hydrogenation of imine **3a**. Various aprotic solvents were evaluated for the asymmetric hydrogenation (entries 7–10). As a result, **4a** was obtained with the highest ee value when the hydrogenation was conducted in an ethereal solvent, such as THF or cyclopentyl methyl ether (CPME). The benzisoxazole was fully converted to the *N*-protected amine **4a** with 44% *ee* after 24 h (entry 11). The hydrogenation of **1a** was conducted by using other amino group protecting agents in place of Boc_2_O. Carboxylic anhydrides, Ac_2_O and Bz_2_O, also worked as the acylating agent in the asymmetric reduction of the benzisoxazole to give the corresponding chiral amine with 43% and 47% *ee*, respectively (entries 12 and 13). Use of sulfonic anhydride resulted in a complex mixture, which contained a small amount of **3a** (entry 14). Various *N*-acylating agents other than acid anhydrides were applied to the asymmetric reactions. No conversion of **1a** took place when the reaction was conducted with Boc-ON (entry 15). Use of *O*-alkoxycarbonyl-*N*-hydroxysuccinimide led to a remarkable increase in the reaction rate and some improvement of the stereoselectivity (entries 16–18). The reactions with Cbz-OSu and Fmoc-OSu in THF gave the desired products **8a** and **9a** with 52% and 56% *ee*, respectively. The hydrogenation product **4a** was formed under 10 atm of hydrogen, but the lower hydrogen pressure caused a significant decrease in the reaction rate (entry 19). Substrate **1a** and imine **3a** completely disappeared from the reaction mixture at 24 h.

**Table 1:** Optimization of reaction conditions for the hydrogenation of **1a***^a^*. 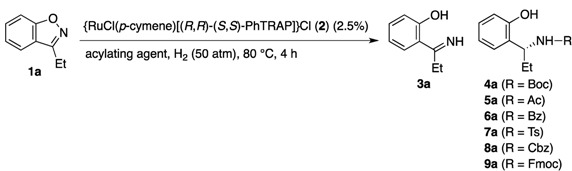

Entry	Solvent	Acylating agent	Yield (3a), % *^b^*	Yield, % *^b,c^*	*ee*, % *^d^*
1	toluene	–	23	0 (**4a**)	–
2	*i*-BuOH	–	12	0 (**4a**)	–
3 *^e^*	toluene	–	9	0 (**4a**)	–
4 *^e^*	*i*-BuOH	–	13	0 (**4a**)	–
5	toluene	Boc_2_O	39	22 (**4a**)	25 (*R*)
6 *^e^*	toluene	Boc_2_O	22	0 (**4a**)	–
7	ClCH_2_CH_2_Cl	Boc_2_O	63	19 (**4a**)	30 (*R*)
8	CPME	Boc_2_O	32	18 (**4a**)	40 (*R*)
9	THF	Boc_2_O	18	31 (**4a**)	39 (*R*)
10	EtOAc	Boc_2_O	22	14 (**4a**)	21 (*R*)
11 *^f^*	THF	Boc_2_O	0	>99 (93) *^g^* (**4a**)	44 (*R*)
12	THF	Ac_2_O	0	87 (**5a**)	43 (*R*)
13	THF	Bz_2_O	0	85 (**6a**)	47 (*R*)
14	THF	Ts_2_O	17	0 (**7a**)	–
15	THF	Boc-ON	0	0 (**4a**)	–
16	CPME	Cbz-OSu	15	58 (**8a**)	43 (*R*)
17	THF	Cbz-OSu	0	>99 (89) *^g^* (**8a**)	52 (*R*)
18	THF	Fmoc-OSu	0	>99 (93) *^g^* (**9a**)	56 (*R*)
19 *^f,h^*	THF	Cbz-OSu	0	82 (**8a**)	52 (*R*)

*^a^* Reactions were conducted on a 0.20 mmol scale in 1.0 mL of solvent. The ratio of **1a**:**2**:acylating agent was 40:1.0:44; *^b^* Determined by ^1^H-NMR analysis of the crude products; *^c^* Yields of **4a**–**9a**. The products are indicated in parentheses; *^d^* Determined by HPLC analysis. The absolute configuration of major enantiomer is indicated in parentheses; *^e^* The reactions were conducted in the presence of TMG (25 mol %). *^f^* The reactions were conducted for 24 h; *^g^* Isolated yields of *N*-protected chiral amines were indicated in parentheses; *^h^* The reactions were conducted under 10 atm of hydrogenation.

Various chiral ligands other than PhTRAP were evaluated for the hydrogenation of **1a** (Table 2). Before the evaluation of ligands, we attempted the catalytic asymmetric reactions by means of a few *in-situ*-generated PhTRAP–ruthenium catalysts. In our previous study on the asymmetric hydrogenation of oxazoles, the chiral catalyst was generated *in situ* from Ru(*η*^3^-methallyl)_2_(cod) and the chiral bisphosphine [30]. However, the chiral ruthenium complex failed to catalyze the conversion of **1a** to imine **3a** (entry 1). No reduction of the C–N double bond was observed in the reaction mixture. The hydrogenation of **1a** was conducted in the presence of the crude ruthenium complex, which was prepared by mixing [RuCl_2_(*p*-cymene)]_2_ and (*R*,*R*)-(*S*,*S*)-PhTRAP in CH_2_Cl_2_–EtOH (1:2) at 50 °C for 1 h and then removing the solvent *in vacuo*. The resulting residue worked as the chiral catalyst as with the isolated complex **2**, akthough its catalyst efficiency was lower than that of **2** (entry 2). By using the procedure for preparing the chiral catalyst from [RuCl_2_(*p*-cymene)]_2_, the hydrogenation of **1a** was conducted with a series of chiral bisphosphines (entries 3–8). As a result of the ligand evaluation, PhTRAP was found to be the most effective ligand for the asymmetric hydrogenation of benzisoxazoles. The ruthenium catalysts prepared from BPPFA [51,52], Josiphos [53], and Chiraphos [54] could cleave the N–O bond in **1a** to give imine **3a** under the hydrogenation conditions, while they are ineffective for the conversion of **3a** into **8a**. Furthermore, the enantiomeric excesses of the chiral products were lower than 20% *ee*. In contrast, [RuCl_2_(*p*-cymene)]_2_–BINAP [55,56] might be competent to reduce the C–N double bond of **3a**, but the catalyst failed to cleave the N–O bond of **1a** with sufficient reaction rate. Furthermore, no conversion of **1a** was observed in the reactions using DIOP [57] and Me-DuPhos ligands [58].

**Table 2:** Effect of chiral catalysts on the hydrogenation of **1a***^a^*. 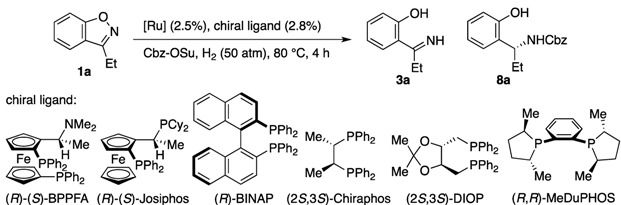

Entry	Chiral ligand	[Ru]	Yield (3a), % *^b^*	Yield (8a), % *^b^*	*ee*, % *^c^*
1 *^d^*	(*R*,*R*)-(*S*,*S*)-PhTRAP	Ru(*η*^3^-methallyl)_2_(cod)	0	0	–
2	(*R*,*R*)-(*S*,*S*)-PhTRAP	[RuCl_2_(*p*-cymene)]_2_	25	47	53 (*R*)
3	(*R*)-(*S*)-BPPFA	[RuCl_2_(*p*-cymene)]_2_	28	6	–
4	(*R*)-(*S*)-Josiphos	[RuCl_2_(*p*-cymene)]_2_	67	15	3 (*R*)
5	(*R*)-BINAP	[RuCl_2_(*p*-cymene)]_2_	0	8	17 (*R*)
6	(2*S*,3*S*)-Chiraphos	[RuCl_2_(*p*-cymene)]_2_	49	10	16 (*S*)
7	(2*S*,3*S*)-DIOP	[RuCl_2_(*p*-cymene)]_2_	0	0	–
8	(*R*,*R*)-Me-DuPhos	[RuCl_2_(*p*-cymene)]_2_	0	0	–

*^a^* Reactions were conducted on a 0.20 mmol scale in 1.0 mL of THF. The ratio of **1a**:chiral ligand:[RuCl_2_(*p*-cymene)]_2_:Cbz-OSu was 40:1.0:0.5:44; *^b^* Determined by ^1^H-NMR analysis of the crude products; *^c^* Determined by HPLC analysis. The absolute configuration of major enantiomer is indicated in parentheses; *^d^* The reaction was conducted with 2.5 mol % of the catalyst precursor.

### 2.2. Asymmetric Hydrogenations of 3-Substituted Benzisoxazoles

As shown in Table 3, the PhTRAP–ruthenium catalyst **2** converted various 3-substituted benzisoxazoles **1** to the corresponding *N*-Cbz-protected *o*-hydroxybenzylamines **8** under the optimized conditions. Reaction rates of the catalytic transformations were affected by the bulkiness of the substituent at the 3-position in **1**. As with **1a**, 3-methylbenzisoxazole **1b** was converted to **8b** with moderate enantiomeric excess (entry 1). Chiral benzylamines **8c** and **8d** were obtained in high yields from the benzisoxazoles bearing 2-phenethyl and isopropyl groups at their 3-position, but complete conversions of **1c** and **1d** needed long reaction times as compared to **1a** or **1b** (entries 2 and 3). Furthermore, the reactions of **1c** and **1d** were less enantioselective. The asymmetric hydrogenation of aryl-substituted substrate **1e** produced the desired chiral diarylamine **8e** with 55% *ee*, but a small amount of achiral diarylmethane was formed through the undesired hydrogenolysis of the benzylic C–N bond (entry 4). The stereoselectivity of the asymmetric reaction may correlate with the electronic property of the substituent at the 5-position of benzisoxazole. Electron-donating groups in **1f** or **1g** slightly improved the enantioselectivity (entries 5 and 6). In contrast, the fluorine atom in **1h** caused a decrease in the ee value of the hydrogenation product (entry 7). The substituent at the 6-position unsystematically affects the stereoselectivity. The reaction of 3,6-dimethylbenzisoxazole (**1j**) proceeded with a comparable stereoselectivity to **1b** (entry 9). However, the selectivity deteriorated in the reaction of the substrate bearing either electron-donating or electron-withdrawing groups at the 6-position (entries 8 and 10). Steric hindrance of the methoxy group in **1l** scarcely affected the yield of the hydrogenation product, but it did cause significant decrease in enantioselectivity (entry 11).

**Table 3:** Catalytic asymmetric hydrogenation of 3-substituted benzisoxazoles **1***^a^*. 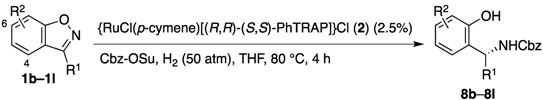

Entry	R^1^	R^2^	Substrate (1)	Product (8)	Yield, % *^b^*	*ee*, % *^c^*
1	Me	H	**1b**	**8b**	78	48
2 *^d^*	CH_2_CH_2_Ph	H	**1c**	**8c**	87	35
3 *^d^*	*i*-Pr	H	**1d**	**8d**	99	40
4 *^d^*	Ph	6-MeO	**1e**	**8e**	74 *^e^*	55
5	Me	5-MeO	**1f**	**8f**	82	54
6	Me	5-Me	**1g**	**8g**	87	57
7 *^d^*	Me	5-F	**1h**	**8h**	76	38
8	Me	6-MeO	**1i**	**8i**	69	40
9	Me	6-Me	**1j**	**8j**	87	51
10 *^d^*	Me	6-F	**1k**	**8k**	82	23
11 *^d^*	Me	4-MeO	**1l**	**8l**	76	25

*^a^* Reactions were conducted on a 0.20 mmol scale in 1.0 mL of THF. The ratio of **1a**:**2**:Cbz-OSu was 40:1.0:44; *^b^* Isolated yield; *^c^* Determined by HPLC analysis; *^d^* The reaction was conducted for 24 h. *^e^* 2-Benzyl-5-methoxyphenol was obtained in 8% yield.

### 2.3. Reaction Pathway of the Asymmetric Hydrogenation of Benzisoxazoles

**Scheme 1:**
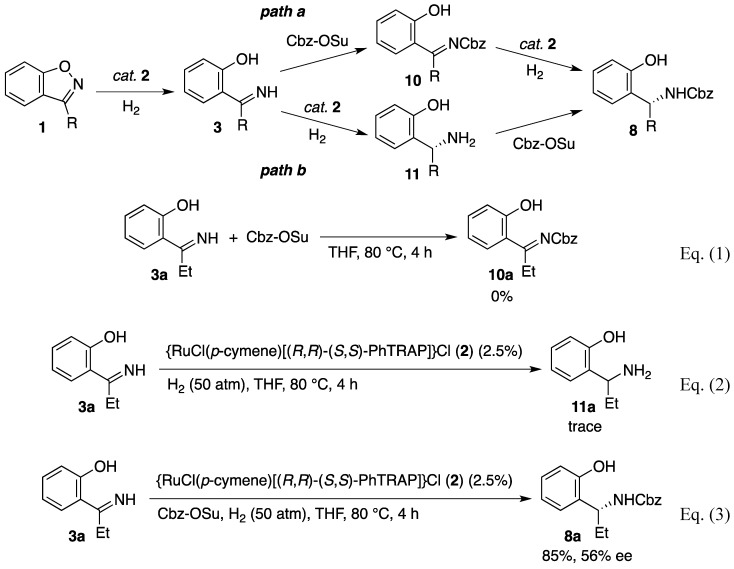
Possible pathways for the asymmetric hydrogenation of benzisoxazoles.

As described above, the hydrogenation of benzisoxazoles **1** proceeds through the reductive N–O bond cleavage to form *o*-hydroxyphenyl imine **3**, yielding *α*-substituted N-Cbz-benzylamine **8** in the presence of Cbz-OSu. The acylating agent is indispensable for the reduction of the imine to amine. Furthermore, the absence of the ruthenium complex prevents the conversion of **1** to **3** as well as the formation of **8**. In light of these observations, two reaction pathways are conceivable for the catalytic asymmetric hydrogenation of benzisoxazoles, as shown in Scheme 1. In both pathways, the ruthenium catalyst **2** initially cleaves the N–O bond in **1**, giving imine **3** [49,50]. One involves the acylation of the imine NH with Cbz-OSu to form intermediate **10** [59,60], which is reduced to **8** by hydrogen through the ruthenium catalysis (path a). In the other possible pathway, the hydrogenation of C–N double bond in **3** occurs prior to the Cbz-protection of the nitrogen atom (path b) [61]. To ascertain these two possibilities, the following reactions were conducted by using the imine **3a** as the substrate. First, the imine **3a** was treated with Cbz-OSu at 80 °C for 4 h in the absence of catalyst **2** (Eq. 1). No formation of *N*-Cbz-imine **10a** was observed in the resulting mixture, and the substrate **3a** remained intact, hence path a can be ruled out. Next, the hydrogenation of **3a** was conducted with the ruthenium catalyst in the absence of Cbz-OSu, but the reaction produced only a small amount of the expected primary amine **11** (Eq. 2). The reduction of the imine **3a**, however, proceeded in the presence of Cbz-OSu, affording **8a** in high yield (Eq. 3). Consequently, the present hydrogenation of the benzisoxazoles should occur through path b. Although the resulting primary amine **11** may strongly inhibit the catalysis of the PhTRAP–ruthenium complex, the generated amino group is rapidly protected by the coexistent Cbz-OSu under the hydrogenation conditions [62,63]. Thus, the rapid acylation effectively avoids the inhibition of the ruthenium catalyst by the free amino group of **11** [64,65].

## 3. Experimental

### 3.1. Materials

Optical rotations and NMR spectra were measured with JASCO P-1020 polarimeter and Bruker AVANCE 400 (9.4 T magnet) spectrometer, respectively. In the ^1^H-NMR spectra, chemical shifts (ppm) were referenced to internal tetramethylsilane (0.00 ppm in CDCl_3_). In ^13^C-NMR spectra, chemical shifts (ppm) were referenced to the carbon signal of the deuterated solvents used (77.0 ppm in CDCl_3_, 30.0 ppm in acetone-*d*_6_). Elemental analyses were performed by Service Centre of Elementary Analysis of Organic Compounds. Flash column chromatographies were performed with silica gel 60 (230–400 mesh, Merck). Benzisoxazole substrates were synthesized from the corresponding *o*-hydroxyphenones through the oxime formation, followed by the *O*-acetylation of the oxime and the intramolecular nucleophilic substitution on the nitrogen atom [66,67]. {RuCl(*p*-cymene)[(*R*,*R*)-(*S*,*S*)-PhTRAP]}Cl was prepared according to the literature procedure [25]. Tetrahydrofuran (THF) was deoxygenated by purging with nitrogen for 30 min and was dried with an alumina and copper column system (GlassContour Co.). Other materials were purchased and used without further purification.

### 3.2. Catalytic Asymmetric Hydrogenation of Benzisoxazoles *1*

General Procedure

Under a nitrogen atmosphere, a benzisoxazole **1** (0.20 mmol) was added to a solution of {RuCl(*p*-cymene)[(*R*,*R*)-(*S*,*S*)-PhTRAP]}Cl (5.5 mg, 5.0 μmol), Cbz-OSu (54 mg, 0.22 mmol) in THF (1.0 mL). The reaction mixture was stirred at 80 °C under 50 atm of hydrogen for 4 or 24 h. The resulting reaction mixture was evaporated under reduced pressure. The residue was purified with a flash column chromatography (EtOAc/hexane) to give the *α*-substituted *N*-Cbz-*o*-hydroxybenzylamine **8**.

*Benzyl (R)-1-(2-hydroxyphenyl)-1-propylcarbamate* (**8a**). Colorless oil, 89% yield, 52% *ee*; [*α*]_D_^26^ = +14.1 (*c* 0.51, CHCl_3_); ^1^H-NMR (400 MHz, CDCl_3_, TMS) δ 0.97 (t, *J* = 7.3 Hz, 3H), 1.93 (quintet, *J* = 7.4 Hz, 2H), 4.77 (q, *J* = 7.8 Hz, 1H), 5.04 (d, *J* = 12.1 Hz, 1H), 5.14 (d, *J* = 12.1 Hz, 1H), 5.14–6.24 (br, 1H), 6.90 (t, *J* = 7.5 Hz, 1H), 6.93 (d, *J* = 7.5 Hz, 1H), 7.15 (d, *J* = 7.9 Hz, 1H), 7.18 (t, *J* = 7.5 Hz, 1H), 7.29–7.38 (m, 5H), 7.68–7.88 (br, 1H); ^13^C {^1^H}-NMR (100 MHz, CDCl_3_, at 50 °C) δ 11.1, 27.5, 52.9 (br), 67.3, 117.3, 120.4, 127.2 (br), 128.1, 128.2, 128.5, 128.6, 136.2, 154.6, 157.5; Anal. Calcd for C_17_H_19_NO_3_: C, 71.56; H, 6.71; N, 4.91. Found: C, 71.43; H, 6.64; N, 4.87.

*Benzyl 1-(2-hydroxyphenyl)-1-ethylcarbamate* (**8b**). Colorless oil, 78% yield, 48% *ee*; [*α*]_D_^26^ = +16.9 (*c* 0.72, CHCl_3_); ^1^H-NMR (400 MHz, CDCl_3_, TMS) δ 1.57 (d, *J* = 6.9 Hz, 3H), 5.04 (d, *J* = 12.1 Hz, 1H), 5.09 (quintet, *J* = 7.5 Hz, 1H), 5.15 (d, *J* = 12.1 Hz, 1H), 5.14–5.24 (br, 1H), 6.89 (t, *J* = 7.5 Hz, 1H), 6.93 (d, *J* = 8.3 Hz, 1H), 7.19 (t, *J* = 7.9 Hz, 1H), 7.20 (d, *J* = 7.7 Hz, 1H), 7.28–7.38 (m, 5H), 7.90–8.10 (br, 1H); ^13^C {^1^H}-NMR (100 MHz, CDCl_3_) δ 20.3, 45.9 (br), 67.4, 117.7, 120.6, 126.3, 128.16, 128.25, 128.5, 128.9, 129.2, 136.1, 154.5, 157.3; Anal. Calcd for C_16_H_17_NO_3_: C, 70.83; H, 6.32; N, 5.16. Found: C, 70.46; H, 6.36; N, 5.18.

*Benzyl 1-(2-hydroxyphenyl)-3-phenyl-1-propylcarbamate* (**8c**). Colorless solid, 87% yield, 35% *ee*; [*α*]_D_^27^ = +8.5 (*c* 1.31, CHCl_3_); ^1^H-NMR (400 MHz, CDCl_3_, TMS) δ 2.22 (q, *J* = 7.7 Hz, 2H), 2.59–2.74 (m, 2H), 4.88 (q, *J* = 7.6 Hz, 1H), 5.05 (d, *J* = 12.1 Hz, 1H), 5.15 (d, *J* = 12.1 Hz, 1H), 5.20-5.36 (br, 1H), 6.87–6.94 (m, 2H), 7.11–7.22 (m, 5H), 7.24–7.38 (m, 7H), 7.61–7.78 (br, 1H); ^13^C {^1^H}-NMR (100 MHz, CDCl_3_, at 50 °C) δ 32.9, 36.1, 50.9 (br), 67.4, 117.5, 120.6, 126.0, 127.2 (br), 128.0, 128.1, 128.2, 128.36, 128.44, 128.5, 128.9, 136.2, 141.2, 154.6, 157.4; Anal. Calcd for C_23_H_23_NO_3_: C, 76.43; H, 6.41; N, 3.88. Found: C, 76.27; H, 6.45; N, 3.90.

*Benzyl 1-(2-hydroxyphenyl)-2-methyl-1-ethylcarbamate* (**8d**). Colorless solid, 99% yield, 40% *ee*; [*α*]_D_^27^ = +12.9 (*c* 1.01, CHCl_3_); ^1^H-NMR (400 MHz, CDCl_3_, TMS) δ 0.80 (d, *J* = 6.6 Hz, 3H), 1.10 (d, *J* = 6.5 Hz, 3H), 2.21 (double septet, *J* = 9.8, 6.6 Hz, 1H), 4.48 (t, *J* = 9.7 Hz, 1H), 5.04 (d, *J* = 12.2 Hz, 1H), 5.13 (d, *J* = 12.2 Hz, 1H), 5.32–5.48 (br, 1H), 6.84–6.91 (m, 2H), 7.02–7.17 (m, 3H), 7.27–7.38 (m, 5H); ^13^C {^1^H}-NMR (100 MHz, CDCl_3_, at 50 °C) δ 19.6, 20.2, 31.9, 58.6 (br), 67.2, 116.9, 120.3, 127.8, 128.0, 128.1, 128.4, 128.5, 136.4, 154.3, 157.4; Anal. Calcd for C_18_H_21_NO_3_: C, 72.22; H, 7.03; N, 4.68. Found: C, 71.95; H, 7.06; N, 4.71.

*Benzyl (2-hydroxy-4-methoxyphenyl)phenylmethylcarbamate* (**8e**). Colorless solid, 74% yield, 55% *ee*; [*α*]_D_^25^ = +30.5 (*c* 0.53, CHCl_3_); ^1^H-NMR (400 MHz, CDCl_3_, TMS) δ 3.76 (s, 3H), 5.11 (d, *J* = 12.1 Hz, 1H), 5.18 (d, *J* = 12.1 Hz, 1H), 5.75 (br d, *J* = 7.6 Hz, 1H), 6.16 (d, *J* = 8.7 Hz, 1H), 6.40 (dd, *J* = 2.4, 8.5 Hz, 1H), 6.46 (s, 1H), 6.85 (d, *J* = 8.5 Hz, 1H), 6.75–7.15 (br, 1H), 7.25–7.37 (m, 10H); ^13^C {^1^H}-NMR (100 MHz, acetone-*d*_6_, at 50 °C) δ 55.3, 55.8, 67.1, 103.2, 106.2, 122.5, 127.6, 128.0, 128.78, 128.81, 129.1, 129.4, 130.5, 138.7, 144.3, 156.6, 157.0, 161.4; Anal. Calcd for C_22_H_21_NO_4_: C, 72.71; H, 5.82; N, 3.85. Found: C, 72.63; H, 5.83; N, 3.79.

*Benzyl 1-(2-hydroxy-5-methoxyphenyl)-1-ethylcarbamate* (**8f**). Colorless oil, 82% yield, 54% *ee*; [*α*]_D_^27^ = +28.2 (*c* 0.99, CHCl_3_); ^1^H-NMR (400 MHz, CDCl_3_, TMS) δ 1.54 (d, *J* = 6.9 Hz, 3H), 3.75 (s, 3H), 5.02 (d, *J* = 12.1 Hz, 1H), 5.05 (quintet, *J* = 7.5 Hz, 1H), 5.14 (d, *J* = 12.1 Hz, 1H), 5.18 (br d, *J* = 7.8 Hz, 1H), 6.72–6.76 (m, 2H), 6.87 (d, *J* = 8.2 Hz, 1H), 7.29–7.38 (m, 5H), 7.52–7.70 (br, 1H); ^13^C {^1^H}-NMR (100 MHz, CDCl_3_, at 50 °C) δ 20.2, 45.9, 55.8, 67.4, 112.4, 113.8, 118.4, 128.16, 128.24, 128.5, 130.3, 136.1, 148.3, 153.8, 157.2; Anal. Calcd for C_17_H_19_NO_4_: C, 67.76; H, 6.36; N, 4.65. Found: C, 67.58; H, 6.21; N, 4.64.

*Benzyl 1-(2-hydroxy-5-methylphenyl)-1-ethylcarbamate* (**8g**). Colorless oil, 87% yield, 57% *ee*; [*α*]_D_^26^ = +27.4 (*c* 0.93, CHCl_3_); ^1^H-NMR (400 MHz, CDCl_3_, TMS) δ 1.55 (d, *J* = 7.0 Hz, 3H), 2.26 (s, 3H), 5.03 (d, *J* = 12.1 Hz, 1H), 5.04 (quintet, *J* = 7.4 Hz, 1H), 5.14 (d, *J* = 12.1 Hz, 1H), 5.17–5.27 (br, 1H), 6.81 (d, *J* = 7.9 Hz, 1H), 6.95–7.00 (m, 2H), 7.25–7.38 (m, 5H), 7.48–7.80 (br, 1H); ^13^C {^1^H}-NMR (100 MHz, CDCl_3_, at 50 °C) δ 20.3, 20.6, 45.9 (br), 67.4, 117.5, 126.8, 128.17, 128.23, 128.5, 129.0, 129.3, 129.8, 136.2, 152.1, 157.2; Anal. Calcd for C_17_H_19_NO_3_: C, 71.56; H, 6.72; N, 4.91. Found: C, 71.23; H, 6.74; N, 4.86.

*Benzyl 1-(5-fluoro-2-hydroxyphenyl)-1-ethylcarbamate* (**8h**). Colorless oil, 76% yield, 38% *ee*; [*α*]_D_^26^ = +6.2 (*c* 1.12, CHCl_3_); ^1^H-NMR (400 MHz, CDCl_3_, TMS) δ 1.55 (d, *J* = 6.9 Hz, 3H), 5.04 (d, *J* = 12.1 Hz, 1H), 5.05 (quintet, *J* = 7.4 Hz, 1H), 5.09–5.17 (br 1H), 5.16 (d, *J* = 12.1 Hz, 1H), 6.86–6.91 (m, 3H), 7.28–7.29 (m, 5H), 7.95–8.20 (m, 1H); ^13^C {^1^H}-NMR (100 MHz, CDCl_3_, at 50 °C) δ 20.1, 45.3 (br), 67.6, 112.4 (d, *J* = 24 Hz), 115.2 (d, *J* = 23 Hz), 118.8 (d, *J* = 7 Hz), 128.2, 128.4, 128.6, 130.6 (d, *J* = 6 Hz), 135.9, 150.5 (d, *J* = 2 Hz), 157.1 (d, *J* = 238 Hz), 157.4; Anal. Calcd for C_16_H_16_FNO_3_: C, 66.43; H, 5.57; N, 4.84. Found: C, 66.20; H, 5.56; N, 4.86.

*Benzyl 1-(2-hydroxy-4-methoxyphenyl)-1-ethylcarbamate* (**8i**). Colorless solid, 69% yield, 40% *ee*; [*α*]_D_^27^ = +14.9 (*c* 0.53, CHCl_3_); ^1^H-NMR (400 MHz, CDCl_3_, TMS) δ 1.55 (d, *J* = 6.9 Hz, 3H), 3.76 (s, 3H), 5.02 (quintet, *J* = 7.4 Hz, 1H), 5.03 (d, *J* = 12.1 Hz, 1H), 5.10–5.19 (br, 1H), 5.15 (d, *J* = 12.1 Hz, 1H), 6.46 (dd, *J* = 2.4, 8.5 Hz, 1H), 6.51 (d, *J* = 2.4 Hz, 1H), 7.09 (d, *J* = 8.5 Hz, 1H), 7.29–7.38 (m, 5H), 8.30–8.50 (br, 1H); ^13^C {^1^H}-NMR (100 MHz, CDCl_3_, at 50 °C) δ 20.3, 45.3 (br), 55.2, 67.4, 103.2, 106.5, 121.7, 126.8, 128.1, 128.2, 128.5, 136.1, 155.8, 157.4, 160.4; Anal. Calcd for C_17_H_19_NO_4_: C, 67.76; H, 6.36; N, 4.65. Found: C, 67.56; H, 6.21; N, 4.56.

*Benzyl 1-(2-hydroxy-4-methylphenyl)-1-ethylcarbamate* (**8j**). Colorless oil, 87% yield, 51% *ee*; [*α*]_D_^26^ = +16.4 (*c* 1.15, CHCl_3_); ^1^H-NMR (400 MHz, CDCl_3_, TMS) δ 1.55 (d, *J* = 7.0 Hz, 3H), 2.28 (s, 3H), 4.99–5.08 (m, 2H), 5.14 (d, *J* = 12.1 Hz, 1H), 5.13–5.25 (br 1H), 6.71 (d, *J* = 7.8 Hz, 1H), 6.74 (s, 1H), 7.08 (d, *J* = 7.8 Hz, 1H), 7.28–7.38 (m, 5H), 7.80–8.10 (br, 1H); ^13^C {^1^H}-NMR (100 MHz, CDCl_3_, at 50 °C) δ 20.3, 20.9, 45.6 (br), 67.4, 118.3, 121.3, 126.1, 126.3, 128.16, 128.23, 128.5, 136.2, 139.0, 154.6, 157.3; Anal. Calcd for C_17_H_19_NO_3_: C, 71.56; H, 6.72; N, 4.91. Found: C, 71.28; H, 6.75; N, 4.87.

*Benzyl 1-(4-fluoro-2-hydroxyphenyl)-1-ethylcarbamate* (**8k**). Colorless oil, 82% yield, 23% *ee*; [*α*]_D_^26^ = –9.6 (*c* 0.69, CHCl_3_); ^1^H-NMR (400 MHz, CDCl_3_, TMS) δ 1.54 (d, *J* = 6.8 Hz, 3H), 5.02 (quintet, *J* = 7.3 Hz, 1H), 5.06 (d, *J* = 12.2 Hz, 1H), 5.16 (d, *J* = 12.2 Hz, 1H), 5.10–5.26 (br, 1H), 6.54–6.64 (m, 2H), 7.12 (t, *J* = 7.4 Hz, 1H), 7.28–7.39 (m, 5H), 8.62–8.74 (br, 1H); ^13^C {^1^H}-NMR (100 MHz, CDCl_3_, at 50 °C) δ 20.3, 45.4 (br), 67.6, 104.9 (d, *J* = 24 Hz), 107.1 (d, *J* = 22 Hz), 125.2 (d, *J* = 3 Hz), 127.0 (d, *J* = 10 Hz), 128.2, 128.4, 128.6, 135.9, 156.1 (d, *J* = 12 Hz), 157.5, 163.0 (d, *J* = 246 Hz); Anal. Calcd for C_16_H_16_FNO_3_: C, 66.43; H, 5.57; N, 4.84. Found: C, 66.23; H, 5.39; N, 4.84.

*Benzyl 1-(2-hydroxy-6-methoxyphenyl)-1-ethylcarbamate* (**8l**). Colorless solid, 76% yield, 25% *ee*; [*α*]_D_^27^ = –0.68 (*c* 0.59, CHCl_3_); ^1^H-NMR (400 MHz, CDCl_3_, TMS) δ 1.52 (d, *J* = 7.0 Hz, 3H), 3.81 (s, 3H), 5.04 (d, *J* = 12.3 Hz, 1H), 5.14 (d, *J* = 12.3 Hz, 1H), 5.39 (dq, *J* = 9.2, 7.0 Hz, 1H), 5.86–5.98 (br, 1H), 6.47 (d, *J* = 8.2 Hz, 1H), 6.49 (d, *J* = 8.1 Hz, 1H), 7.07 (t, *J* = 8.3 Hz, 1H), 7.24–7.38 (m, 5H); ^13^C {^1^H}-NMR (100 MHz, CDCl_3_, at 50 °C) δ 20.6, 43.2, 55.7, 66.9, 103.5, 109.8, 117.8, 128.0, 128.2, 128.5, 136.7, 154.9, 156.9, 158.1; Anal. Calcd for C_17_H_19_NO_4_: C, 67.76; H, 6.36; N, 4.65. Found: C, 67.65; H, 6.29; N, 4.68.

## 4. Conclusions

In this study, we proved that 3-substituted benzisoxazoles **1** react with hydrogen in the presence of the chiral ruthenium catalyst, {RuCl(*p*-cymene)[(*R*,*R*)-(*S*,*S*)-PhTRAP]}Cl (**2**). The ruthenium-catalyzed hydrogenation proceeds in the presence of an acylating agent such as Boc_2_O and Cbz-OSu, to afford *α*-substituted *N*-protected *o*-hydroxybenzylamines **4**–**9** in high yields with moderate enantioselectivities. The conversion of **1** to the chiral amines proceeds through the imine intermediate **3**, which is generated from the reductive cleavage of the N–O bond in the benzisoxazole ring. The C–N double bond of **3** is hydrogenated in moderate enantioselectivity by the PhTRAP–ruthenium catalyst, however, the resulting primary amine **11** causes deactivation of the catalyst **2**. The deterioration of **2** can be avoided by the coexistent acylating agent, which rapidly reacts with the amino group.

Although the asymmetric hydrogenation of **1** with PhTRAP–ruthenium catalyst proceeds with moderate stereoselectivity at the current moment, various 3-substituted benzisoxazoles are transformed into the chiral *o*-hydroxybenzylamines in high yields. The products contain a molecular framework, which is often used as a chiral source in asymmetric synthesis and seen in various biologically active compounds. Consequently, the present catalytic asymmetric reaction may be potentially useful for organic synthesis. Further improvement of the stereoselectivity is in progress.
